# Nasal tissue-derived hamartoma in the maxillary gingiva of a calf

**DOI:** 10.1186/s12917-016-0637-4

**Published:** 2016-01-22

**Authors:** Takeshi Tsuka, Takehito Morita, Hinako Tanaka, Shinji Kono, Yusuke Murahata, Kazuo Azuma, Tomohiro Osaki, Norihiko Ito, Yoshiharu Okamoto, Tomohiro Imagawa

**Affiliations:** Department of Veterinary Clinical Medicine, School of Veterinary Medicine, Faculty of Agriculture, Tottori University, 4-101, Koyama-Minami, Tottori, Tottori Japan; Tobu Veterinary Clinic, Tottori Prefectural Federation Agricultural Mutual Aid Association, 210-19, Takadote, Kuniyasu, Tottori, Tottori Japan

**Keywords:** Calf, Computed tomography, Hamartoma, Maxillary gingiva, Nasal tissue

## Abstract

**Background:**

All of oral hamartomas has been previously found in mandibular gingiva in younger calves, and were histologically diagnosed as a vascular hamartoma. This is the first case report describing a calf with a mass in the maxillary gingiva that was histologically diagnosed as a nasal tissue-derived hamartoma.

**Case presentation:**

A 13-day-old male Holstein calf presented with a horn-like mass in the left rostral maxillary gingiva. Surgical removal revealed that the mass had a dual structure composed of cartilaginous and soft tissues and extended deeply toward the nasal cavity. Excised tissues mainly consisted of two types of mature cells without mitotic figures and atypia: 1) the cartilage-like structures consisted of an island and a meandering massive focus of mature cartilaginous tissues, and 2) tubular structures consisting of stratified ciliated mucosal columnar cells with gland-like structures and aggregated goblet cells. The mass was diagnosed as a nasal tissue-derived hamartoma because these two types of structures were histologically identical to nasal structures. The present case had no recurrence at 1 year after surgery.

**Conclusions:**

To our knowledge, this is the first description of the calf with nasal tissue-derived hamartoma in the maxillary gingiva.

## Background

Hamartomas have been defined as excessive focal overgrowths of mature cells in organs of identical cellular elements [1, 2]. Most hamartomas are present at birth and differ from true tumors by showing growth coordinated with the surrounding tissues [2]. In bovines, the tissues of such congenital lesions arise from various organs and are classified as vascular [1–4], fibrous [5], and/or bronchiolar and alveolar structures [6]. Gingival hamartomas have occasionally been reported in younger calves [1–4], and have been grossly characterized as flat, reddish masses located on the mandibular gingiva [1–4]. All such lesions have been histologically diagnosed as vascular hamartomas [1–4]. The present report describes the specific gross appearance and histological findings of a mass observed in the maxillary gingiva of a newborn calf, which differed from those of previous gingival vascular hamartomas.

## Case presentation

**Fig. 1:**
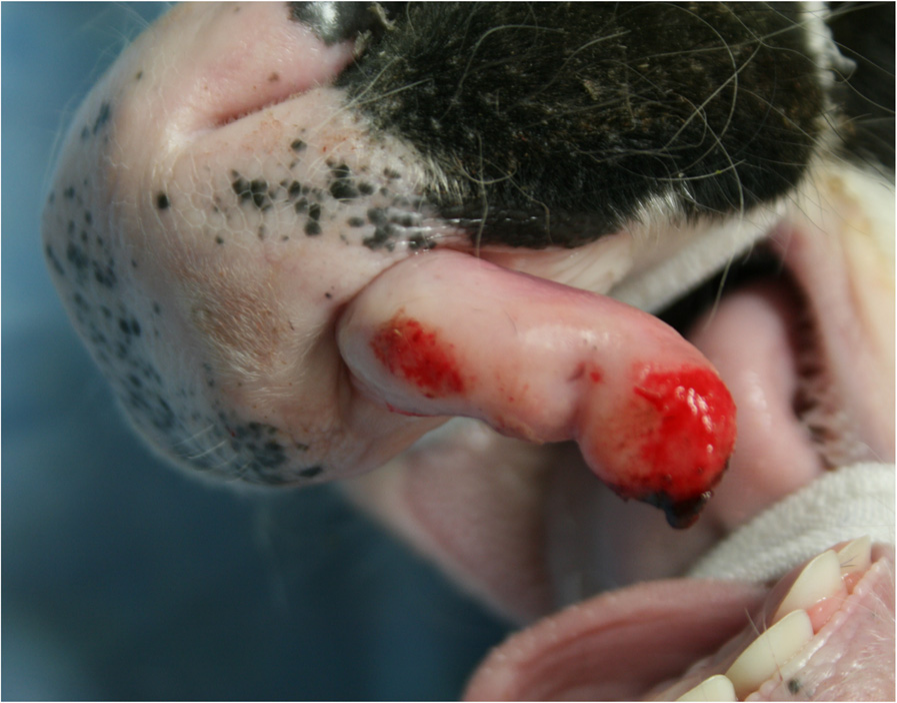
Gross appearance of the mouth in a 13-day-old male Holstein calf. The calf has a horn-like and 3.5-cm-height mass, which is colored similar to the gingival mucosa, in the left rostral maxillary gingiva

**Fig. 2:**
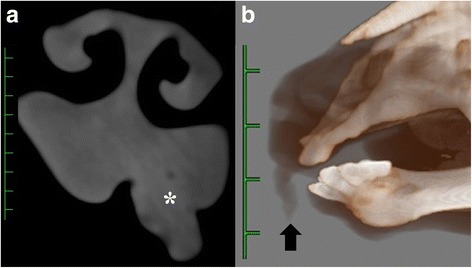
Axial computed tomographic image (**a**) and three-dimensional computed tomographic image visualizing the soft tissues and bones of the face (**b**) in a 13-day-old male Holstein calf. **a** The mass (asterisk) possesses anatomical continuity with the nasal structures. Scale: 10 mm. **b** The mass (arrow) arises cranial than the rostal edge of the maxillary bone. Scale: 25 mm

A 13-day-old male Holstein calf presented with a horn-like mass in the left and rostral maxillary gingiva (Fig. 1). The calf had good weight gain, and showed normal eating and drinking behavior, although a small amount of hemorrhage was found near the top of the mass during eating. Respiratory signs including cough, and dyspnea were not evident. The horn-like mass was 1.5 cm in width and 3.5 cm in height, and was colored similar to the gingival mucosa. On palpation, the mass was firmly fixed with the deeper structures. Palpation did not reveal whether the mass was derived from maxillary bones, although a hard structure could be felt inside of the mass. Under sedation with intravenous xylazine hydrochloride (0.01 mg/kg), the calf was examined by computed tomography (CT) scanning using a helical CT device (Pronto SE, Hitachi Co. Ltd, Tokyo, Japan) for preoperative observation of the positional relationship of the mass with deeper structures (the nose and maxillary bones). The mass was demonstrated to be a homogenous and slightly bright gray structure than the neighboring soft tissues within the root of the mass, and included no radiopaque structure (i.e., bone or dent) (Fig. 2a). Neither osteolysis nor ossification was evident in the maxillary bone near the mass. Three-dimensional (3D) CT revealed that the mass arose cranial than the rostal edge of the maxillary bone and possessed anatomical continuity with the nasal structures (Fig. 2b). The mass was removed from the basal region by cutting along the gingival mucosa with an electric scalpel soon after the CT examination. The mass had a dual structure comprised of white cartilaginous tissues and soft tissues surrounding a cartilaginous core. The root of the mass extended into the deeper site of the maxillary gingiva toward the nasal cavity, and was removed as deeply as possible. The operative opening was covered by suturing the gingival mucosa over the opening with a monofilament absorbable suture. Follow-up one year after surgery found no recurrence.

**Fig. 3:**
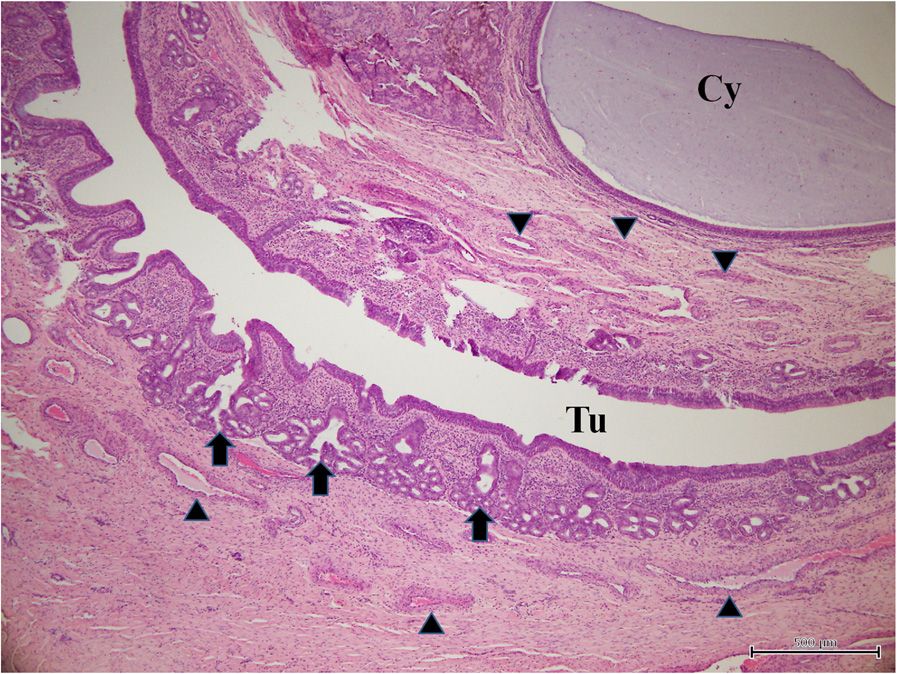
Histology of the nasal tissue-derived hamartoma. The cystic structures are surrounded by epithelial cells. Tubular structures are surrounded by stratified ciliated mucosal columnar cells, gland-like structures, and arterial and venous vessels. Arrowheads: arterial and venous vessels. Black arrows: gland-like structures. Cy: cystic structures. Tu: tubular structures. Hematoxylin and eosin (H&E) stain. Bar = 500 μm

**Fig. 4:**
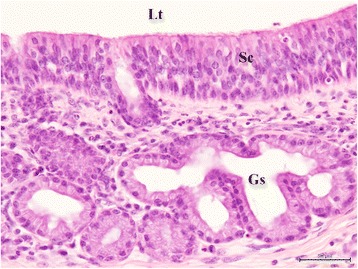
Histology of the nasal tissue-derived hamartoma. The tubular structures are lined by stratified ciliated mucosal columnar cells and gland-like structures. Lt: Lumen of tubules. Sc: stratified ciliated mucosal columnar cells. Gs: gland-like structures. Hematoxylin and eosin (H&E) stain. Bar = 50 μm

**Fig. 5:**
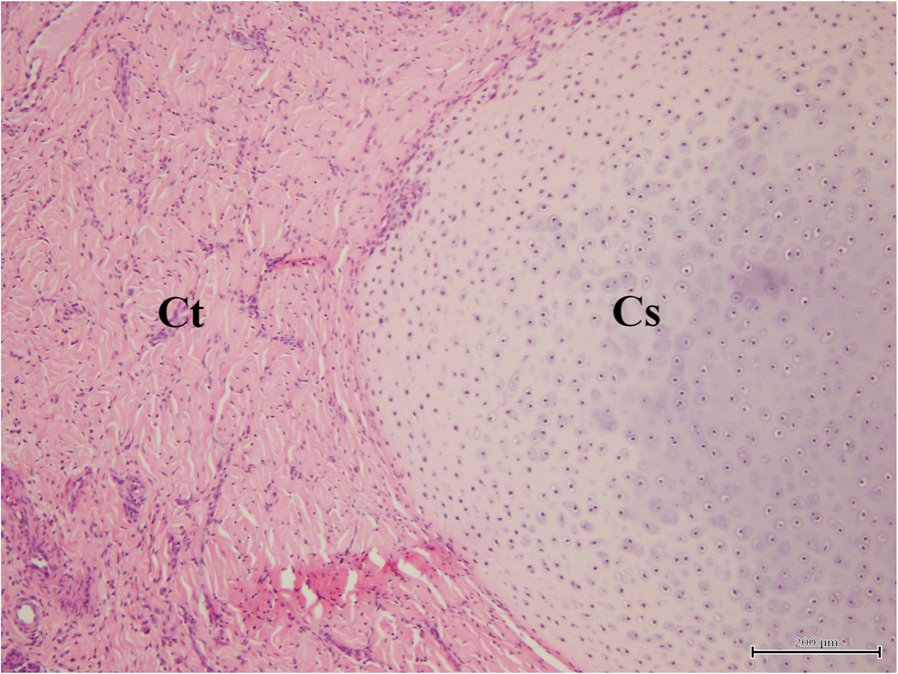
Histology of the nasal tissue-derived hamartoma. The cartilage-like structures are surrounded by collagen tissues. Cs: cartilage-like structures. Ct: collagen tissues. Hematoxylin and eosin (H&E) stain. Bar = 200 μm

The mass was composed of white cartilaginous tissues and soft tissues reaching from the basal part to the tip of the horn-like mass on the cut surface. The excised tissue was fixed in 10 % neutral buffered formalin, and embedded in paraffin wax. Sections were cut at a thickness of 5 micrometer, and stained with haematoxylin and eosin (H&E). The surface structures of the mass were lined with stratified squamous mucosal cells with gland-like nasal structures. Two types of luminal structures composed of cystic and tubular structures were seen in deeper areas of the surface structures (Fig. 3). The cystic structures were surrounded by epithelial cells, and possibly originated from tubular structures. Tubular structures lined with stratified ciliated mucosal columnar cells were surrounded by arterial and venous vessels, and gland-like structures that contained basophilic mucinoid materials (Fig. 4). Within the tubular structures, some goblet cells were occasionally aggregated in the mucosal cell layer, and lymphocytes and plasma cells frequently infiltrated beneath the mucosa. The cartilage-like structures, which were randomly observed near cystic and tubular structures, were compsed of an island and a meandering massive focus of mature cartilaginous tissues, and were surrounded by collagenous tissues (Fig. 5). Mitotic figures and atypic cells were not found among the overall structures of the mass.

Gingival hamartomas have been occasionally reported in calves ranging in age from 1 day to 2.5-months [1–4], and were diagnosed as vascular hamartomas based on pathological findings of large numbers of disorganized capillaries lined by mature endothelial cells [1]. The pathological findings of the present case can be summarized as follows: 1) the mass was comprised of cartilage-like structures, and tubular structures consisting of stratified ciliated mucosal columnar cells and gland-like structures; 2) the cellular composition in the cartilage-like and tubular structures of the mass were identical to those commonly found in nasal cavities; and 3) all structures were formed by proliferation of normal mature cells without mitotic abnormalities. Based on these histological findings, the mass was diagnosed as a nasal tissue-derived hamartoma.

There are large differences in the gross appearance of gingival vascular hamartomas versus nasal tissue-derived hamartoma. Gingival vascular hamartomas are characterized as reddish-colored flat masses (2–7 cm in widths and 1.5 cm in heights), whereas nasal tissue-derived hamartoma are a horn-like mass (1.5 cm in width and 3.5 cm in height) colored similar to the gingival mucosa [1–4]. Moreover, nasal tissue-derived hamartomas in the maxillary gingiva have different locations than gingival vascular hamartomas, which have been found in the rostral levels of the mandibular gingiva [1–4]. The cause of this difference may be that the presently described lesion originated from nasal structures and protruded to the oral cavity.

Hamartomas derived from nasal structures have been described in humans and cats [7–11]. Most human and feline patients with various types of nasal hamartomas have presented with upper respiratory signs, and the lesions were observed within the nasal cavity [7–11]. However, there are reported cases in which the lesions were first discovered during the course of a dental examination [7], which resembles the present case showing a clinical pattern of a nasal lesion that invaded the oral cavity. In human patients, nasal hamartomas have been reported to be predominantly composed of mesenchymal tissues (called as nasal chondromesenchymal hamartomas) or epithelial tissues (called as respiratory epithelial adenomatoid hamartomas) [8]. Nasal chondromesenchymal hamartomas are characterized by a mixture of mesenchymal elements (spindle cells, and collagen fibers) and irregular islands of osseous and chondroid tissues [8]. Respiratory epithelial adenomatoid hamartomas are characterized by formation of large and small adenomatoid structures lined by ciliated respiratory epithelium [7].

The present case was likely to have pathological findings identical to a mixture of these two types of lesions. Some human patients have been reported to have a respiratory epithelial adenomatoid hamartoma component together with an additional chondro-osseous component [9]. In veterinary medicine, nasal hamartomas have been reported in six feline cases: one case was diagnosed as a nasal vascular hamartoma and five cases were diagnosed as nasal mesenchymal hamartomas [10, 11]. Feline nasal mesenchymal hamartoma is diagnosed based on the pathological finding of the tissues mainly comprised of various mesenchymal components including cartilage, woven bone, and spindle cells [10]. Pathological findings of these lesions would be similar to those of nasal chondromesenchymal hamartomas in humans [8]. Nasal epithelial lesions identical to respiratory epithelial adenomatoid hamartomas in humans have not previously been reported in veterinary medicine. The present case may be the first reported animal case presenting with a mixed-type nasal hamartoma comprised of both epithelial and mesenchymal tissues.

## Conclusions

To our knowledge, this is the first description of a calf with a nasal tissue-derived hamartoma in the maxillary gingiva. Nasal tissue-derived hamartoma is an unusual lesion pathologically identical to a combination of nasal chondromesenchymal hamartoma and respiratory epithelial adenomatoid hamartoma in veterinary species.

## Ethical statement

All diagnostic and therapeutic procedures were performed by the approved veterinarians in the course of routine veterinary health management.
